# Supplementary material for: Multichannel Acoustic Spectroscopy of the Human Body for Inviolable Biometric Authentication
Source: Biosensors (Basel). 2022 Aug 31;12(9):700. doi: 10.3390/bios12090700 (PMC9496529; doi:10.3390/bios12090700)
Supplement: Supplementary file 1 [file biosensors-12-00700-s001.zip › biosensors-1837312-supplementary.pdf]

Supplementary Materials

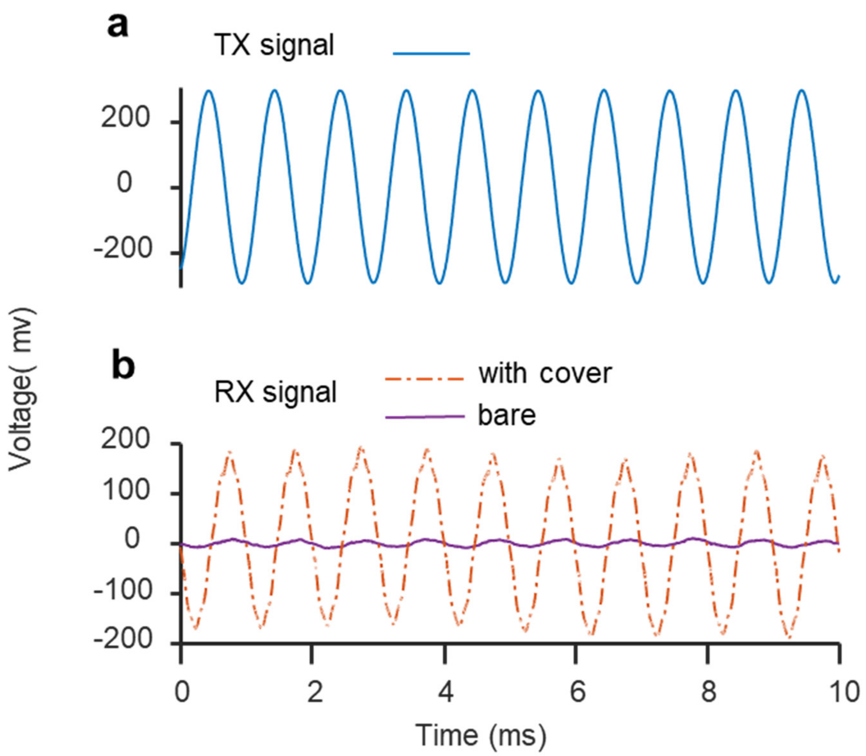

Supplementary Figure S1. Effect of PDMS impedance matching layer. (a) The shape of the acoustic impedance spectrum applied to a finger through a bone conduction transducer. (b) The difference in the signal received from the microphone depending on whether the microphone is covered with a polydimethylsiloxane. TX: transmitted, RX: received.

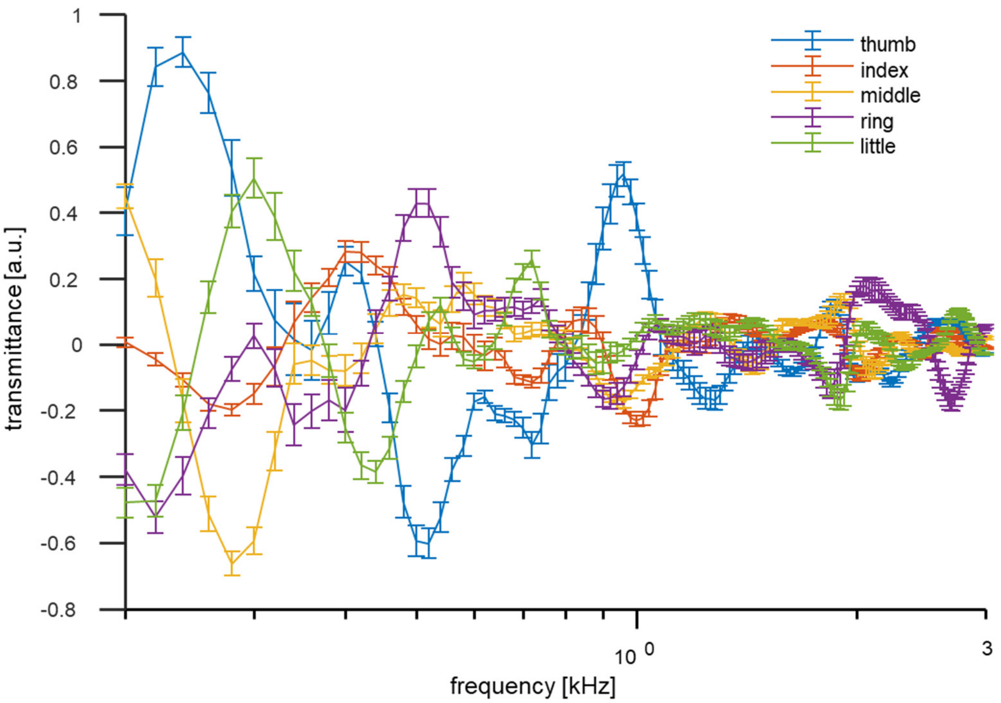

Supplementary Figure S2. Acoustic spectra of each finger (thumb, index, middle, ring, and little) of the subject up to 3 kHz. The error bars in the figure indicate the relative standard deviation.

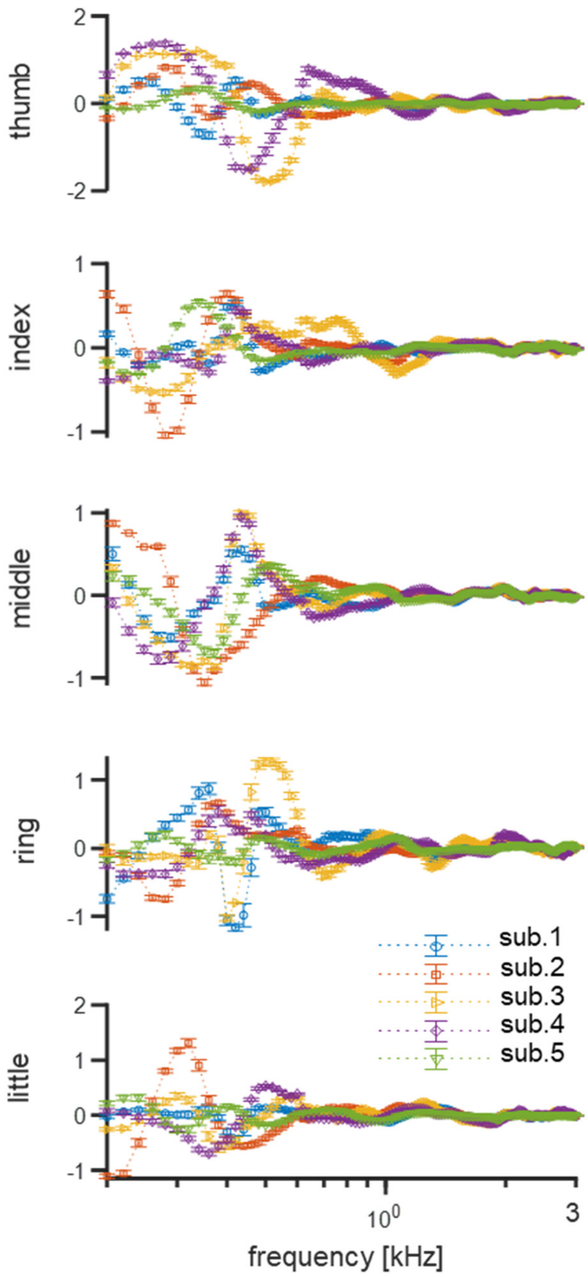

Supplementary Figure S3. Finger acoustic spectra up to 3kHz of five subjects for assessing interpersonal variation. The error bars in the figure indicate the relative standard deviation.

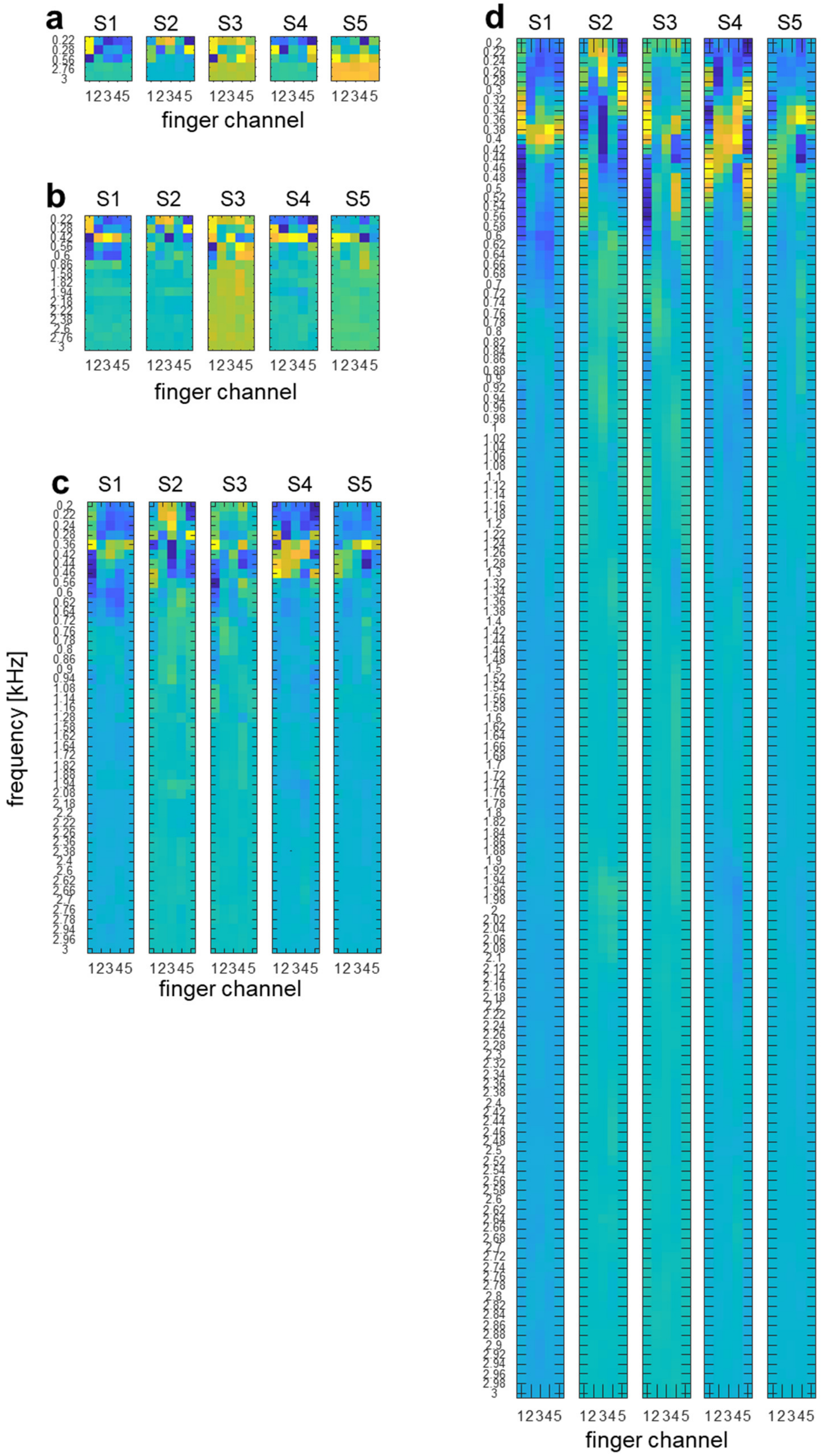

Supplementary Figure S4. 2D intensity images of five finger channel acoustic spectra of five subjects for four different numbers of selected frequencies: (a) 5, (b) 15, (c) 47, and (d) 141.

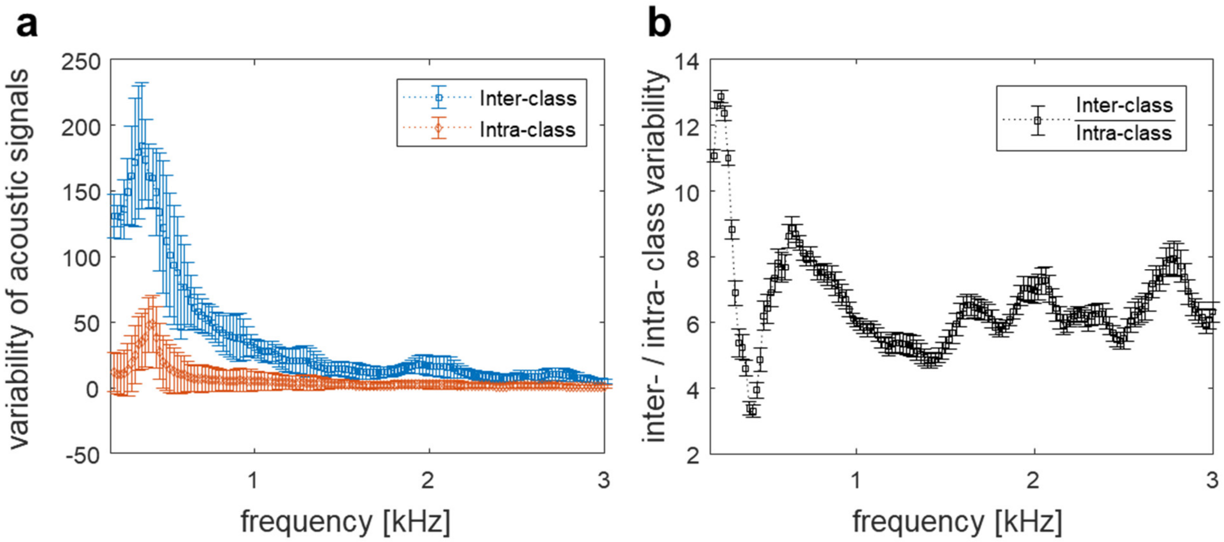

Supplementary Figure S5. The variability of transmitted acoustic signal amplitude for each frequency. (a) Intra-class variability and inter-class variability for each frequency. (b) Quantitative pattern obtained by dividing the calculated inter-class variation by intra-class variation for each frequency.

SUPPLEMENTARY TABLE S1

COMPARISON OF PERFORMANCE FOR FOUR MODELS ACCORDING TO THE NUMBER OF CHANNELS

Classifier	*Number of Channels*				
	1	2	3	4	5
RF	79.13±1.51	92.60±0.20	96.71±0.20	98.45±0.10	99.08
kNN	68.06±0.93	83.09±0.36	89.13±0.23	92.13±0.54	92.20
LDA	75.41±1.93	89.65±0.28	94.61±0.54	96.31± 0.12	96.64
SVM	64.79±1.26	80.28±0.26	86.83±0.28	89.92± 0.75	93.27

SUPPLEMENTARY TABLE S2

IDENTIFICATION ACCURACY IN THE RF MODEL ACCORDING TO FINGER CHANNEL COMBINATION

Ch	Combination	Accuracy [%]	*Ch*	Combination	Accuracy [%]
1	*1*	76.68	*3*	*1, 2, 3*	97.32
	2	78.86		*1, 2, 4*	96.79
	3	80.83		*1, 2, 5*	96.71
	4	79.45		*1, 3, 4*	96.75
	5	80.02		*1, 3, 5*	96.68
2	*1, 2*	92.58		*1, 4, 5*	96.56
	1, 3	92.85		*2, 3, 4*	96.57
	1, 4	92.50		*2, 3, 5*	96.63
	1, 5	92.14		*2, 4, 5*	96.61
	2, 3	92.65		*3, 4, 5*	96.58
	2, 4	92.43	*4*	*1, 2, 3, 4*	98.39
	2, 5	92.68		*1, 2 ,3, 5*	98.32
	3, 4	92.68		*1, 2, 4, 5*	98.11
	3, 5	92.80		*1, 3, 4, 5*	98.24
	4, 5	92.74		*2, 3, 4, 5*	98.23
			5	*1, 2, 3, 4, 5*	99.08

SUPPLEMENTARY TABLE S3

AVERAGE CLASSIFICATION ACCURACY OF EACH FREQUENCY SECTION

Freq. sections [kHz]	Mean accuracy [%]	*Freq. sections [kHz]*	Mean accuracy [%]
0 – 0.3	96.99	*1.5 – 1.8*	92.72
0.3 – 0.6	94.60	*1.8 – 2.1*	93.38
0.6 – 0.9	92.53	*2.1 – 2.4*	94.12
0.9 – 1.2	91.73	*2.4 – 2.7*	93.24
1.2 – 1.5	91.91	*2.7 – 3.0*	94.31

SUPPLEMENTARY TABLE S4

CLASSIFICATION ACCURACY WITH THE INCREASING NUMBER OF FREQUENCIES

N. of freq.	Acc. [%]	*N. of freq.*	Acc. [%]	*N. of freq.*	Acc. [%]	*N. of freq.*	Acc. [%]
1	39.30	*36*	99.46	*71*	99.54	*106*	99.39
2	70.49	*37*	99.54	*72*	99.54	*107*	99.39
3	83.49	*38*	99.54	*73*	99.62	*108*	99.39
4	89.14	*39*	99.54	*74*	99.54	*109*	99.39
5	92.89	*40*	99.54	*75*	99.62	*110*	99.39
6	94.42	*41*	99.54	*76*	99.54	*111*	99.39
7	95.80	*42*	99.54	*77*	99.62	*112*	99.39
8	96.79	*43*	99.54	*78*	99.54	*113*	99.31
9	97.63	*44*	99.54	*79*	99.62	*114*	99.31
10	97.94	*45*	99.54	*80*	99.62	*115*	99.39
11	98.47	*46*	99.54	*81*	99.54	*116*	99.31
12	98.62	*47*	99.62	*82*	99.46	*117*	99.39
13	98.78	*48*	99.54	*83*	99.62	*118*	99.31
14	99.01	*49*	99.62	*84*	99.54	*119*	99.31
15	99.08	*50*	99.62	*85*	99.54	*120*	99.24
16	99.08	*51*	99.62	*86*	99.46	*121*	99.31
17	99.24	*52*	99.62	*87*	99.46	*122*	99.16
18	99.31	*53*	99.62	*88*	99.46	*123*	99.24
19	99.46	*54*	99.62	*89*	99.54	*124*	99.16
20	99.46	*55*	99.62	*90*	99.46	*125*	99.16
21	99.46	*56*	99.54	*91*	99.54	*126*	99.16
22	99.46	*57*	99.54	*92*	99.54	*127*	99.08
23	99.54	*58*	99.54	*93*	99.54	*128*	99.16
24	99.46	*59*	99.62	*94*	99.54	*129*	99.08
25	99.39	*60*	99.54	*95*	99.46	*130*	99.08
26	99.39	*61*	99.62	*96*	99.46	*131*	99.08
27	99.39	*62*	99.62	*97*	99.46	*132*	99.08
28	99.46	*63*	99.62	*98*	99.39	*133*	99.08
29	99.46	*64*	99.54	*99*	99.39	*134*	99.24
30	99.46	*65*	99.54	*100*	99.46	*135*	99.08
31	99.46	*66*	99.54	*101*	99.46	*136*	99.08
32	99.46	*67*	99.62	*102*	99.46	*137*	99.08
33	99.54	*68*	99.54	*103*	99.46	*138*	99.16
34	99.54	*69*	99.54	*104*	99.39	*139*	99.08
35	99.54	*70*	99.54	*105*	99.39	*140*	99.08
